# Analogue Hawking Radiation as a Tunneling in a Two-Level PT-Symmetric System

**DOI:** 10.3390/e25081202

**Published:** 2023-08-12

**Authors:** Bijan Bagchi, Rahul Ghosh, Sauvik Sen

**Affiliations:** Department of Physics, Shiv Nadar Institution of Eminence, Gautam Buddha Nagar, Greater Noida 203207, Uttar Pradesh, India; rg928@snu.edu.in (R.G.); sauviksen.physics@gmail.com (S.S.)

**Keywords:** Hawking radiation, PT symmetry, tunneling probability, exceptional points

## Abstract

In light of a general scenario of a two-level non-Hermitian PT-symmetric Hamiltonian, we apply the tetrad-based method to analyze the possibility of analogue Hawking radiation. We carry this out by making use of the conventional null-geodesic approach, wherein the associated Hawking radiation is described as a quantum tunneling process across a classically forbidden barrier on which the event horizon imposes. An interesting aspect of our result is that our estimate for the tunneling probability is independent of the non-Hermitian parameter that defines the guiding Hamiltonian.

## 1. Introduction

The physics of black holes has continually aroused interest after Bekenstien–Hawking’s pioneering works in the 1970s, in which the authors tried to interpret them as thermodynamical objects that release radiation outside their event horizon [1,2,3,4] (see, for a review of the literature, [5]). The idea of Hawking radiation exploits the concept of the creation of pair production next to the event horizon (out of the vacuum), with one of the particles running away to infinite space from the boundary, while the other with negative energy is sucked into the black hole, resulting in a decrease in mass until the whole black hole disappears in a cloud of radiation. A natural question has been asked as to whether viable information could be gathered at temperatures near the scale of Planckian mass when the quantum gravitational effects become substantial [6].

In this paper, our primary focus is to look at a suitable structure of a non-Hermitian two-level effective Hamiltonian [7,8] to illustrate the possibility of artificial Hawking radiation. We carry this out by mapping to a coordinate setting and making use of the tetrad-based method. The latter strategy is frequently used for seeking solutions pertaining to a curved space. We will demonstrate that black hole similarities emerge with the emission of Hawking-like radiation when the event horizon causes the separation of two distinct topological regions [9,10].

A non-interacting field theory is often sought to address Hawking radiation. In fact, it was observed that in two-dimensional Schwarzschild geometry, interaction effects are minor, and a free particle theory is adequate for the treatment of Hawking radiation; see for some detailed discussion [11,12]. In the following, we adopt the procedure of Parikh and Wilczek to estimate the tunneling probability by employing the standard classical approach of WKB approximation [13]. In their formalism, the effect of the back-reaction was included to ensure energy conservation while a particle was emitted through the process of tunneling when moving past the horizon. Noting that the need for a nonsingular coordinate system is essential at the horizon, we adopt below the well-known Painleve–Gullstrand coordinates [14,15], which are simply coordinate transformations of the usual Schwarzschild solution. The corresponding metric tensor, which involves an off-diagonal element, is regular at the Schwarzschild radius but has a singularity only at the origin; see, for example, [16]. It needs to be mentioned that prior to the work of [13], the idea of Hawking radiation as tunneling was first investigated by Srinivasan and Padmanabhan [17], including a treatment of different coordinate settings [18].

However, a remark is in order. The need to use Painlevé–Gullstrand coordinates for the Schwarzschild metric arises due to the singularity in the usual Schwarzschild coordinates. However, the use of Kruskal–Szekeres coordinates was shown to yield an incorrect formulation [19,20], implying that one should be able to use other coordinates so long as one does not have a real singularity instead of a coordinate singularity.

## 2. Biorthogonality and Exceptional Point

The most general 2×2 Hermitian Hamiltonian (with the Fermi velocity set to unity) in terms of the linear node $\vec{k}=\left(k_{x},k_{y},k_{z}\right)$ has the form [21,22] ${\hat{h}}_{0}=\chi \left(k\right)+\sum_{i=x,y,z}d_{i}\left(k\right)\sigma_{i}$, where χ and $d_{i}$ are suitable real coefficients, $k=|\vec{k}|$, and the $\sigma_{i}$ variables are Pauli matrices. The Hamiltonian that characterizes the conduction and valence bands supports a pair of eigenvalues whose separation is removed when the coefficients vanish simultaneously. This is at once obvious from the energy dispersion providing the energy splitting

(1) $$\epsilon_{\pm }=\chi \pm \sqrt{d_{x}^{2}+d_{y}^{2}+d_{z}^{2}}$$

We see that degeneracy requires all three $d_{i}$s to become zero together, i.e., $d_{x}=d_{y}=d_{z}=0$, which is not symmetry-protected. In Weyl semimetals, in which the conduction and valence bands’ energies coincide over a certain region of the Brillouin zone, a linear crossing of two bands takes place at the forming of nondegenerate Dirac cones corresponding to the conduction and valance bands.

The extension of topological phases from the Hermitian to the non-Hermitian sector has been pursued in a variety of papers [23], and the presence of gains and losses has been investigated [24,25]. A point was made a few years ago about the question of whether real black holes can emit Hawking radiation and whether meaningful information can be gathered about Planckian physics [6]. Very recently, De Beule et al. [26] made an explicit analysis of the existence of artificial event horizons in Weyl semimetal heterostructures. In their work, the electronic analogs of stimulated Hawking emissions was studied, and physical observables were identified. Sabsovich et al. [27] examined black and white hole analogs in Weyl semimetals subjected to inhomogeneous nodal tilts, explored experimentally viable consequences, and showed the general relativity analogy of such Hamiltonians. An analogy was also drawn in some papers between the low-energy Hamiltonian of tilted nodes and black hole metrics [8,28,29]. The possibility of the emission of Hawking radiation was investigated in a somewhat similar context [30]. Further, an imitation of black hole Hawking radiation was found in a purely classical-mechanical system by employing a coupled double-chain model admitting frequency dispersion [31]. The tied-up issue of the tunneling probability across the event horizon points was explored in the framework of a two-level non-Hermitian topologically insulated Weyl-type Hamiltonian, which contained titing in one of the directions [32].

A two-dimensional structure of a non-Hermitian dissipative Hamiltonian was also featured in an elaborate investigation of analogue Schwarzschild black holes emitting Hawking radiation [33]. A typical situation is described by the inclusion of a non-Hermitian term $i\vec{\tau }\left(k\right)\cdot \vec{\sigma }$, with $\vec{\tau }=\left(\tau_{x},\tau_{y},\tau_{z}\right)$, to ${\hat{h}}_{0}$ when the overall Hamiltonian assumes the form $\hat{h}={\hat{h}}_{0}+i\vec{\tau }\left(k\right)\cdot \vec{\sigma }$. In such a situation, the eigenvalues are

(2) $$E_{\pm }\left(k\right)=\chi \pm \sqrt{∆}$$

where $∆=d_{x}^{2}+d_{y}^{2}+d_{z}^{2}-\left(\tau_{x}^{2}+\tau_{y}^{2}+\tau_{z}^{2}\right)+2i\left(\tau_{x}d_{x}+\tau_{y}d_{y}+\tau_{z}d_{z}\right)$. A consequence is that these become equal when ∆=0, pointing to the presence of an exceptional point [34,35,36,37,38] where we have

(3) $$d_{x}^{2}+d_{y}^{2}+d_{z}^{2}=\tau_{x}^{2}+\tau_{y}^{2}+\tau_{z}^{2}\;\mathrm{and}\;\tau_{x}d_{x}+\tau_{y}d_{y}+\tau_{z}d_{z}=0$$

Defining the right and left eigenstates as

(4) $$\begin{array}{c} \hat{h}\left(k\right)|\psi_{\pm }^{R}\rangle =E_{\pm }|\psi_{\pm }^{R}\rangle \end{array}$$

(5) $$\begin{array}{c} {\hat{h}}^{†}\left(k\right)|\psi_{\pm }^{L}\rangle =E_{\pm }^{*}|\psi_{\pm }^{L}\rangle \end{array}$$

where $|\psi_{\pm }^{R}\rangle$ and $|\psi_{\pm }^{R}\rangle$ are explicitly

(6) $$\begin{array}{c} |\psi_{\pm }^{R}\rangle =\frac{1}{\sqrt{2\left(E_{\pm }-\chi \right)\left(E_{\pm }-\chi +d_{z}+i\tau_{z}\right)}}{\left[E_{\pm }-\chi +d_{z}+i\tau_{z},\;\left(d_{x}+id_{y}\right)-\left(\tau_{y}-i\tau_{x}\right)\right]}^{T} \end{array}$$

(7) $$\begin{array}{c} |\psi_{\pm }^{L}\rangle =\frac{1}{\sqrt{2\left(E_{\pm }^{*}-\chi \right)\left(E_{\pm }^{*}-\chi +d_{z}-i\tau_{z}\right)}}{\left[E_{\pm }^{*}-\chi +d_{z}-i\tau_{z},\;\left(d_{x}+id_{y}\right)+\left(\tau_{y}-i\tau_{x}\right)\right]}^{T} \end{array}$$

the biorthogonality relations are a ready outcome.

(8) $$\begin{array}{c} \langle \psi_{\alpha }^{L}|\psi_{\beta }^{R}\rangle =\delta_{\alpha \beta }\;\alpha ,\beta =\pm \end{array}$$

The self-orthogonality of the eigenstates can be worked out easily [39].

## 3. A Two-Level PT-Symmetric Model

### 3.1. The Hamiltonian

Consider the following arrangement of the *d*-coefficients to enquire into the spectral phase transition as the system transits to exhibiting complex eigenvalues from the real ones:

(9) $$d_{x}=\rho \left(k\right)\cos\phi \left(k\right),\;d_{y}=\rho \left(k\right)\sin\phi \left(k\right),\;d_{z}=0,\;\tau_{x}=\tau_{y}=0,\;\tau_{z}=\lambda \eta \left(k\right)$$

where *k* is a real variable, ρ(k),η(k), and ϕ(k) are a set of real, nonzero periodic functions, and $\lambda \in {ℜ}^{+}$ is a coupling parameter. The inclusion of the latter implies the introduction of gain and loss in the system, thereby signaling the possibility of the appearance of exceptional points where abrupt phase transitions could occur. The class of representations seen in (9) have also been studied in [40,41].

Expressed in matrix form, the Hamiltonian corresponding to (9) reads

(10) $$\hat{h}\to \hat{H}=\left(\begin{array}{cc} T(k) & S(k)\\ S^{*}\left(k\right) & T^{*}\left(k\right) \end{array}\right),\;\hat{H}\neq {\hat{H}}^{†}$$

where T=χ(k)+iλη(k) and $S=\rho \left(k\right)e^{i\phi (k)}$. The Hermitian counterpart of $\hat{H}\left(k\right)$ corresponds to k=0. Enforcing PT symmetry [42] requires the diagonal elements of the matrix generated by (10) to be complex conjugates of each other, and this is similarly true for the off-diagonal elements as well. $\hat{H}\left(k\right)$ is easily seen to commute with the joint operator PT, i.e., $[\hat{H},\mathrm{PT}]=0$, where the P operator is represented by $\sigma_{x}$ and T stands for the usual complex conjugation operation.

Apart from analytical evaluations [43], numerical algorithms for the diagonalization of PT-symmetric Hamiltonians have also been carried out in the literature [44]. The possibility of PT symmetry residing in quantum mechanical systems is a prominent forefront in research. Their position was soon found to be intermediate between open and closed systems. While the role of non-Hermiticity in understanding stable phases has been pursued in the literature for the last several years [45,46], the character of PT symmetry for stable nodal points concerning gapped and gapless semimetals [22,47], where the invariants are constituted of Bloch bands, is a somewhat recent realization. Indeed, due to such a symmetry prevailing, one finds stable nodal points to exist in lesser dimensions [48].

The eigenvalues of $\hat{H}$ are easily seen to satisfy the relation

(11) $$E_{\pm }\left(k\right)=\chi \left(k\right)\pm \eta \left(k\right)\sqrt{{\bar{\lambda }}^{2}\left(k\right)-\lambda^{2}},\;\bar{\lambda }\left(k\right)\equiv \frac{\rho (k)}{\eta (k)}$$

Introducing $\tan\theta =\frac{\rho (k)}{i\lambda \eta (k)}$, where θ=θ(k), one can classify the accompanying right eigenvectors as

(12) $${\left[\cos\left(\frac{\theta }{2}\right),\;\sin\left(\frac{\theta }{2}\right)e^{-i\phi }\right]}^{T},\;{\left[\sin\left(\frac{\theta }{2}\right),\;-\cos\left(\frac{\theta }{2}\right)e^{-i\phi }\right]}^{T}$$

while their left partners are

(13) $${\left[\cos^{*}\left(\frac{\theta }{2}\right),\;\sin^{*}\left(\frac{\theta }{2}\right)e^{-i\phi }\right]}^{T},\;{\left[\sin^{*}\left(\frac{\theta }{2}\right),\;-\cos^{*}\left(\frac{\theta }{2}\right)e^{-i\phi }\right]}^{T}$$

We can readily check that these obey the biorthogonal conditions (8).

Expression (11) clearly shows that the eigenvalues stay real when the inequality $\lambda <\bar{\lambda }$ holds. This corresponds to the situation when PT is unbroken. However, when the opposite is the case, i.e., $\lambda >\bar{\lambda }$, a broken PT phase is encountered. At the critical value $\lambda =\bar{\lambda }$, exceptional points appear when both the eigenvalues $E_{+}$ and $E_{-}$ coincide to become χ(k) and the associated eigenvectors coalesce to form a single entity. In other words, at the exceptional points, we have ρ=±λη.

It is worthwhile to mention that a simpler form of $\hat{H}$, proposed by Bender et al. [49] a few years ago, discusses the type $\hat{H}=q\cos\phi ¶+iq\sin\phi \sigma_{z}+s\sigma_{x}$, which supports the eigenvalues $\lambda_{\pm }=q\cos\phi \pm \sqrt{s^{2}-q^{2}\sin^{2}\phi }$. These remain entirely real when the inequality $s^{2}>q^{2}\sin^{2}\phi$ is obeyed.

### 3.2. The Tetrad Representation

Let us represent the Hamiltonian in the following tetrad basis:

(14) $$\hat{H}=e{}^{\mu }{}_{a}h_{\mu }\sigma^{a}+e{}^{\mu }{}_{0}h_{\mu }¶$$

Let us also assume that all $h_{\mu }$ variables are suitable entities to be matched. The vielbeins $e{}^{\mu }{}_{a}$ and $e{}^{\mu }{}_{0}$ satisfy the orthonormality conditions $e{{}^{a}}_{\mu }e{{}^{\mu }}_{b}=\delta {{}^{a}}_{b}$, μ=(0,x,y,z), and a,b=(x,y,z) subject to the metric being expressed as the bilinear combination of the tetrads

(15) $$g^{\mu \nu }=e{}^{\mu }{}_{\alpha }e{}^{\nu }{}_{\beta }\;\eta^{\alpha \beta }$$

where $\eta^{\alpha \beta }=diag\left(-1,\;1,\;1,\;1\right)$ is the Minkowski metric of flat spacetime.

To proceed with (15), let us first write the vielbeins $e_{a}$${}^{\mu }$ in terms of four functions, $f_{1},f_{2},g_{1},$ and $g_{2}$, and let us then seek to relate them with the known functions at hand, namely χ(k),ρ(k),η(k), and ϕ(k). A convenient set of vielbeins is given by [50]

(16) $$e_{0}{}^{0}=f_{1},\;e_{1}{}^{0}=f_{2},\;e_{0}{}^{1}=g_{2},\;e_{1}{}^{1}=g_{1},\;e_{2}{}^{2}=\frac{1}{r},\;e_{3}{}^{3}=\frac{1}{r\sin\theta }$$

with the respective inverses

(17) $$\begin{array}{c} e^{0}{}_{0}=\frac{g_{1}}{f_{1}g_{1}-f_{2}g_{2}},\;e^{0}{}_{1}=-\frac{f_{2}}{f_{1}g_{1}-f_{2}g_{2}},\;e^{1}{}_{0}=-\frac{g_{2}}{f_{1}g_{1}-f_{2}g_{2}},\;\\ e^{1}{}_{1}=\frac{f_{1}}{f_{1}g_{1}-f_{2}g_{2}},\;e^{2}{}_{2}=r,\;e^{3}{}_{3}=r\sin\theta \end{array}$$

The line element then simplifies to the form

(18) $$ds^{2}=\frac{g_{1}^{2}-g_{2}^{2}}{f_{1}g_{1}^{2}}dt^{2}+\frac{2g_{2}}{f_{1}g_{1}^{2}}dtdr-\frac{1}{g_{1}^{2}}dr^{2}-r^{2}d\Omega^{2}$$

The Schwarzschild gauge arises for the following choice of $f_{1},f_{2},g_{1},$ or $g_{2}$, namely

(19) $$f_{1}=1,\;f_{2}=0,\;g_{1}=1,\;g_{2}=-\sqrt{\frac{2M}{r}}$$

and yields the following form of the black hole metric in Painlevé–Gullstrand coordinates:

(20) $$ds^{2}=\left(1-\frac{2M}{r}\right)dt^{2}-2\sqrt{\frac{2M}{r}}drdt-dr^{2}-r^{2}d\Omega^{2}$$

where M is the mass of the black hole and *t* represents the Painlevé time. Metric (20) is stationary (i.e., invariant under the translation of *t*) but not static (i.e., not invariant under time reversal) and is consistent with the transformation originally proposed in [14,15].

With the help of (14) and (16), the general form of the Hamiltonian emerges as

(21) $$\begin{array}{c} \hat{H}=\frac{g_{1}h_{0}-g_{2}h_{1}}{f_{1}g_{1}-f_{2}g_{2}}¶+\frac{-f_{2}h_{0}+f_{1}h_{1}}{f_{1}g_{1}-f_{2}g_{2}}\sigma_{x}+rh_{2}\sigma_{y}+r\sin\theta h_{3}\sigma_{z} \end{array}$$

Compared with (18), we can easily derive the coordinate-space correspondence

(22) $$\begin{array}{c} \chi \left(k\right)\to \frac{g_{1}h_{0}-g_{2}h_{1}}{f_{1}g_{1}-f_{2}g_{2}},\;\rho \left(k\right)\cos\phi \to \frac{-f_{2}h_{0}+f_{1}h_{1}}{f_{1}g_{1}-f_{2}g_{2}},\;\rho \left(k\right)\sin\phi \to r,\;\lambda \eta \left(k\right)\to r\sin\theta \end{array}$$

where we have specified $h_{0}=h_{1}=h_{2}=1$ and $h_{3}=i$. Using the values in (19), we have the mapping correspondence to the (r,θ) variables,

(23) $$\begin{array}{c} \chi \to 1+\sqrt{\frac{2M}{r}},\;\rho \cos\phi \to 1,\;\rho \sin\phi \to r,\;\lambda \eta \to r\sin\theta \end{array}$$

which is consistent with $\rho \to \sqrt{r^{2}+1}$ and $\phi \to \tan^{-1}\left(r\right)$. As a result, the Hamiltonian assumes the form

(24) $$\hat{H}=\left(1+\sqrt{\frac{2M}{r}}\right)¶+\sigma_{x}+r\sigma_{y}+ir\sin\theta \sigma_{z}$$

In the following we estimate the probability transmission amplitude of the analogue Hawking radiation by making use of the correspondence set up in (23).

## 4. Analogue Hawking Radiation and Tunneling Estimate

First of all, the energy eigenvalues acquired read

(25) $$E_{\pm }=\left(1+\sqrt{\frac{2M}{r}}\right)\pm \sqrt{1+r^{2}\cos^{2}\theta }$$

when using (11). The exceptional points correspond to r=±isecθ, which is located on the imaginary axis. In what follows, we will adhere to the positive sign. We then have

(26) $$dE=\frac{dM}{\sqrt{2Mr}}$$

Before we calculate the tunneling probability, let us note that when the particle escapes from the black hole with an energy ω, the mass of the black hole decreases from M to M−ω. Indeed, as pair production takes place around the event horizon, the positive energy particle, when breaking free [13], has to transit through the separating region defined between $r_{in}$, which is the radius of the black hole before the emission of the particle, and $r_{out}$, which is the radius of the black hole after the emission of the particle and which acts as a possible barrier wall. This is possible if the particle can tunnel through such a barrier. Actually, a classically inaccessible zone is replicated if the particle possesses energy below such a resistance.

In dealing with the tunneling problem, we observe that since the action ζ in the transmission region is imaginary, the probability of tunneling taking place can be straightforwardly calculated by making use of the semiclassical WKB approximation [13]. Here, an s-wave particle is considered to go outwards from $r_{in}$ to $r_{out}$, meaning that ζ can be cast in the form

(27) $$Im\zeta =Im\int_{r_{in}}^{r_{out}}p_{r}dr=Im\int_{r_{in}}^{r_{out}}\int_{0}^{p_{r}}dp_{r}dr$$

where the equation of motion for the canonical momentum is imposed as prescribed by the classical Hamilton equation. Noting that the Hamiltonian assumes the respective values M and M−ω for $p_{r}=0$ and $p_{r}=p_{r}$, Imζ can be transformed to [51]

(28) $$Im\zeta =-Im\int_{r_{in}}^{r_{out}}\int_{0}^{\omega }\frac{d\omega }{\dot{r}}dr$$

where, since the emitted particle has a very negligible mass, we can approximate $M=M_{in}\approx M_{out}$ and dM≈−dω.

For the metric at hand, the presence of the horizon can be determined from the radial null geodesic condition $ds^{2}=0$ corresponding to (20). The resulting differential equation becomes

(29) $${\dot{r}}^{2}+2\sqrt{\frac{2M}{r}}\dot{r}-\left(1-\frac{2M}{r}\right)=0$$

which admits the following acceptable solution:

(30) $$\dot{r}=1-\sqrt{\frac{2M}{r}}$$

Substituting in (28) results in

(31) $$Im\zeta =-Im\int_{2M}^{2(M-\omega )}\int_{0}^{\omega }\frac{d\omega dr}{1-\sqrt{\frac{2(M-\omega )}{r}}}$$

which can be reduced to a tractable form using the residue theorem with the help of the substitution $\alpha^{2}=\frac{2(M-\omega )}{r}$. This yields

(32) $$Im\zeta =-Im\int_{2M}^{2(M-\omega )}dr\int_{0}^{\omega }\frac{d\alpha }{1-\alpha }=4\pi \omega$$

Extracting the imaginary part from the right side, for the tunneling probability, we obtain the result

(33) $$\Gamma \approx e^{-2\;Im\zeta }=e^{-8\pi \omega }$$

A similar estimate was made in [13] while accounting for global conservation laws. It is to be noticed that Imζ as given by (33) is independent of the parameters of the model Hamiltonian. Also, it is important to point out that we do not claim invariance under the canonical transformation of the tunneling rate as given by (20), as also follows from the works of [19,20]. Furthermore, for the inclusion of the temporal contribution to the tunneling rate, we refer to the works in [52,53].

In conclusion, concerning the observable that could be associated with Hawking radiation, a natural question arises as to what is the observable that would prove to be analogous to the Hawking radiation and, in particular, the decay rate of complex eigenstates. Indeed, because of the near impossibility of observing Hawking radiation in a real black hole [54], seeking black-hole analogues has become an interesting alternative because these could reveal properties akin to gravitational black holes, with the emission of Hawking-like radiation being a specific one. In this regard, the construction of experimental set-ups has been undertaken to identify the quantum fluctuations that might emerge [55]. Also, very recently, the observation of stimulated Hawking radiation was reported, which occurs in a regime of extreme nonlinear fiber optics [56]. Another point that requires more detailed study is the relationship between the decay rates addressed in [57,58,59] and the rate estimated in (33). As a final remark, let us state that we can formally extend our model to other black hole metrics, such as the charged black holes in 2D dilaton gravity, which originates from the low-energy effective theory of type 0A string theory [60], as well as for a rotating and charged black hole background [61].

## 5. Summary

Non-Hermitian Hamiltonians are found to play a central role in diverse physical problems. In this paper, we studied a generalized form of a two-level PT-symmetric system that depends on a real parameter k and exhibits a period of 2π. Adopting the approach of tetrad formalism, a correspondence was established between such a Hamiltonian and the one constructed in terms of vielbeins. This enabled us to connect to the metric of curved spacetime. By suitably writing the tetrad components in terms of four unknown functions and specifically choosing them such that the Schwrarzschild metric could be described in Painlevé-Gullstrand coordinates, we were able to create a one-to-one correspondence with our chosen form of the Hamiltonian. We computed the probability transmission amplitude of Hawking radiation by looking at it as a tunneling process and making use of the semiclassical WKB approximation. Our result turned out to be independent of the non-Hermitian parameter λ, so the nature of the phase transitions that the system supports does not influence the estimated tunneling probability.

## Data Availability

All the data supporting the findings of this study are included in the article.

## Abbreviations

The following abbreviations are used in this manuscript:

PT	Parity–time operator
